# A Simple Radioassay to Detect Nanoscale Membrane Disruption

**DOI:** 10.3390/mps6020023

**Published:** 2023-02-25

**Authors:** Neha Nanajkar, Lekhana S. Mruthyunjaya, Deepesh Nagarajan

**Affiliations:** 1 Department of Biology, University of Maryland, College Park, MD 20742, USA; 2 Department of Food Technology, M.S. Ramaiah University of Applied Sciences, Bangalore 560054, India; 3 Department of Biotechnology, M.S. Ramaiah University of Applied Sciences, Bangalore 560054, India; 4 Department of Microbiology, St. Xavier’s College, Mumbai 400001, India

**Keywords:** radioassay, membrane damage, antimicrobial peptides, colistin, antibiotics

## Abstract

Understanding the mechanisms and kinetics of membrane damage is of interest to researchers in several overlapping fields of biology. In this study, we describe the development and validation of a simple ${}^{32}$PO${}_{4}^{3-}$ release radioassay used to track nanometer-scale damage to the bacterial cell membrane. Nanoscale membrane damage will result in the release of small cytoplasmic molecules, such as amino acids, sugars, and osmolytes. Our radioassay tracks the release of these molecules using the release of cytoplasmic ${}^{32}$PO${}_{4}^{3-}$ as a proxy. Our assay can both detect ${}^{32}$PO${}_{4}^{3-}$ release and track release kinetics in the order of minutes. We demonstrate the use of our radioassay using *A. baumannii* treated with colistin and Ω76: two agents known to cause membrane damage. Our assay tracks greater membrane damage in *A. baumannii* treated with both these agents, compared to an untreated control. Our assay fills a niche that is not covered by traditional ${}^{51}$Cr release radioassays and fluorescent staining techniques. Furthermore, our assay can potentially be used to track membrane damage in other membrane systems such as lipid vesicles, animal cells, and organelles.

## 1. Introduction

The cell membrane is a prerequisite for life [1]. Life exists because the cell membrane concentrates biomolecules and separates them from the outside environment. Life ceases to exist when the cell membrane is irreparably damaged. Understanding the cell membrane and the mechanisms of its disruption is therefore a topic of interest across several fields, including immunology, apoptosis biology, cancer biology, and antibiotic discovery. Several methods for detecting membrane damage have been developed, but they can be classified into three major categories: radiometry, fluorometry, and microscopy.

The ${}^{51}$Cr release radioassay is used to detect membrane damage via a simple procedure: target cells are labeled with ${}^{51}$Cr; cytolysis of the target cells results in membrane damage, releasing ${}^{51}$Cr; the degree of cytolysis can then be quantified by measuring the radioactivity of the medium [2]. ${}^{51}$Cr release radioassays are considered to be the gold standard for measuring the cell-mediated cytotoxicity of T-cells and natural killer (NK) cells co-cultured with target cells [3]. ${}^{51}$Cr assays have been used to detect cytotoxic reactions to rat Schwann cells [4], the recognition of influenza-infected cells by T-cells [5], and the phagocytic killing of *Candida albicans* [6].

However, the ${}^{51}$Cr release radioassay has three major limitations. Firstly, it can only produce one reading at the end of the assay and therefore cannot measure kinetics [2,3]. Secondly, ${}^{51}$Cr is a γ-emitter [7].Working with ${}^{51}$Cr requires lead shielding and careful dose monitoring [8]. This has lead to the gradual phasing out of ${}^{51}$Cr release radioassays in favor of newer fluorescence and bioluminescence techniques [9]. Thirdly, ${}^{51}$CrO${}_{4}^{2-}$ binds to bacterial lipopolysaccharides on the outer membrane of bacteria [10]. While useful for assaying cell death, ${}^{51}$Cr cannot be used to assay damage to the inner membrane.

The propidium iodide fluorescence assay has emerged as a popular alternative to the ${}^{51}$Cr release radioassay [11,12,13]. The principle of fluorescence assays is simple: propiduim iodide present in the media will only enter dead cells via large perforations in the cell membrane. Once inside, the dye intercalates with DNA and fluoresces [14]. This dye will not enter and stain living cells. Intracellular propidum iodide can then be tracked using flow cytometry [15] or fluorescence microscopy [16]. The fluorescent dyes annexin-V [17] and 7-amino-actinomcin D [18] may be used as alternatives to propidium iodide.

Large-scale membrane damage can directly be observed using scanning electron microscopy (SEM) [19,20,21], transmission electron microscopy (TEM) [22], cryo-electron microscopy (cryo-EM) [23], or atomic force microsopy (AFM) [24,25,26]. However, such microscopic techniques are qualitative in nature and require sophisticated instruments to perform.

In this work, we describe a simple radioassay to detect membrane disruption via the formation of nanometer-scale pores using ${}^{32}$PO${}_{4}^{3-}$ as a tracer. We had previously used this assay to characterize Ω76 [27], an antimicrobial peptide. We have now described the detailed protocol for use by the scientific community. Here, ${}^{32}$PO${}_{4}^{3-}$ is introduced into the bacterial cytoplasm via passive diffusion and is released upon the action of membrane disrupting agents. Unlike ${}^{51}$Cr, ${}^{32}$P is a β-emitter. Working with ${}^{32}$PO${}_{4}^{3-}$ only requires acrylic shielding. Further, our radioassay is capable of tracking ${}^{32}$PO${}_{4}^{3-}$ release kinetics in the order of minutes, and if required, seconds. We believe the assay described here will be of use to bacteriologists studying membrane disruption kinetics and can potentially be applied to any other membrane system as well.

## 2. Experimental Design

### 2.1. Materials

#### 2.1.1. Radiolabeled Phosphate (${}^{32}$PO${}_{4}^{3-}$)

This item can be purchased from any vendor. However this study used 25 μCi/μL ${}^{32}$PO${}_{4}^{3-}$ purchased from BRIT India (catalogue number: LCP-32).

#### 2.1.2. Membrane-Disrupting Agents

This study used Colistin sulfate salt (Sigma C4461-100MG, lot no. SLBT0851, St. Louis, MO, USA) and Ω76 (synthesized by Genscript Inc., Hong Kong, China) to disrupt bacterial cell membranes; Ω76 may also be purchased from NovoPro Bioscience Inc. (catalogue number: 318759) or requested from the authors. You may test any known or putative membrane disrupting agent using this protocol. However, we recommend using Ω76 as a positive control.

#### 2.1.3. Bacterial Culture

This study tested membrane disrupting agents against *A. baumannii* (P1270). This culture can be purchased from the Microbial Type Culture Collection (MTCC), Chandigarh (MTCC culture number: 12889). You may test a known membrane disrupting agent against any bacterial or eukaryotic cell culture.

#### 2.1.4. Culture Media

Mueller Hinton broth was purchased from Sigma/Merck (catalogue number: 70192-100G). We prepared 0.8% physiological saline using NaCl (generic) and Milli-Q water.

#### 2.1.5. Glassware and Plasticware

A 100 mL glass/plastic conical flask, 1.5 mL microcentrifuge tubes (Eppendorfs), 5 mL microcentrifuge tubes (Eppendorfs), 10 mL or 50 mL centrifuge tubes (Falcons), 20 mL syringes with needles, 0.2 μm syringe filters, micropipettes, and tips of all appropriate volumes are required.

### 2.2. Equipment

#### 2.2.1. Scintillation Counter

This study used a Perkin-Elmer MicroBeta 2 2450 Microplate counter.

#### 2.2.2. Radiation Protection

An acrylic radiation shield, appropriate personal protective equipment (PPE), and a Geiger–Müller counter are needed while handling ${}^{32}$PO${}_{4}^{3-}$. Store, handle, and discard radioisotopes as per your institutional guidelines. *The Practical Radiation Technical Manual* (IAEA) [28] provides detailed instructions on precautions needed while handling radioisotopes. In the event of a radiation spill, stop work immediately, notify personnel in the area of the spill, clean the spill with absorbent paper while wearing disposable gloves, dispose of your gloves and absorbent paper into the radioisotope waste container, survey yourself and the area to ensure that radiation levels have dropped to background levels, and inform your radiation safety officer (RSO) before resuming work.

#### 2.2.3. Cold Room (4 ${}^{\circ }$C)

All steps in this protocol need to be performed in a cold room to keep the cells being assayed metabolically inactive. Alternatively, an ice bath may be used for all steps following ${}^{32}$PO${}_{4}^{3-}$ uptake.

#### 2.2.4. Incubator-Shaker

Our incubator-shaker was set at 37 ${}^{\circ }$C/180 rpm.

#### 2.2.5. Centrifuge

The centrifuge must be capable of reaching speeds of at least 12,000 rpm and with rotors to accommodate 1.5 mL and 5 mL microcentrifuge tubes. Note that 5 mL microcentrifuge tubes may be substituted with 10 or 50 mL centifuge tubes if the appropriate rotor is unavailable.

#### 2.2.6. Gel-Rocker

A gel-rocker is required for the gentle rocking of cells to aid the passive diffusion of ${}^{32}$PO${}_{4}^{3-}$.

#### 2.2.7. Aseptic Environment

A laminar flow hood or bunsen burner is required to create an aseptic environment while inoculating your culture. An aseptic environment is not required for further steps in this protocol.

## 3. Protocol

### 3.1. Radiolabeled Phosphate Uptake

Note that ${}^{32}$PO${}_{4}^{3-}$ is very easily introduced into the bacterial cytoplasm via passive diffusion after incubation for 24 h. Care must be taken to incubate your culture at 4 ${}^{\circ }$C to suspend bacterial metabolism and prevent the incorporation of phosphate into biomolecules.

1. Inoculate your culture in 10 mL of Muller Hinton broth. Incubate at 37 ${}^{\circ }$C/24 h, on a shaker incubator at 180 rpm.
2. Pipette 2 mL of this culture into a suitable container (preferably a 5 mL microcentrifuge tube) and centrifuge at 10,000 rpm for 10 min. Collect the pellet and discard the supernatant.
3. Resuspend the pellet with 2 mL fresh Muller Hinton broth (tube A1). 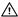
**NOTE**: Fresh broth is essential for ${}^{32}$PO${}_{4}^{3-}$ uptake.
4. Add 100 µCi ${}^{32}$PO${}_{4}^{3-}$ to tube A1. 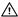
**CAUTION**: Place an acrylic radiation shield between you and the radiation source whenever handling radioisotopes. Wear appropriate PPE.
5. Incubate tube A1 on a gel rocker at 4 ${}^{\circ }$C for 24 h. The ${}^{32}$PO${}_{4}^{3-}$ uptake occurs via passive diffusion across the cell membrane in metabolically inactive cells.

**Figure 1:**
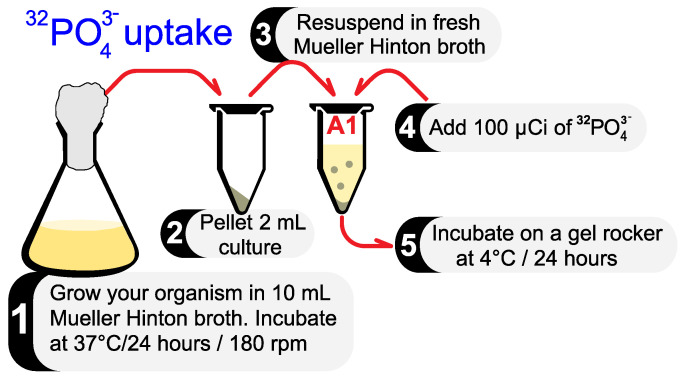
All the steps required to allow a bacterial culture to passively uptake ${}^{32}$PO${}_{4}^{3-}$.

All the steps described above are illustrated in Figure 1.

### 3.2. Radiolabeled Phosphate Retention Check

After incubation, it is essential to verify that ${}^{32}$PO${}_{4}^{3-}$ entered, and is firmly retained within, the bacterial cytoplasm. This can be confirmed using a series of washing and pelleting steps.

1. Pipette 500 μL of the incubated culture in tube A1 into an empty centrifuge tube (tube A2). The remaining culture in tube A1 can be refrigerated and used for further experiments.
2. Centrifuge tube A2 at 12,000 rpm for 5 min at 4 ${}^{\circ }$C. Separate the pellet (tube P1) and supernatant (tube S1).
3. Resuspend P1 in 500 μL physiological saline. 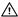
**NOTE**: Do not use phosphate-buffered saline at any step in this protocol. Unlabeled phosphate may compete with radiolabeled phosphate.
4–9. Repeat Steps 2–3 three more times. Over the course of this protocol, your pellet should be resuspended in physiological saline four times (P1→P4), resulting in four centrifuge tubes containing different supernatants at every step of the washing process (S1→S4).
10. Use a scintillation counter to enumerate the disintegration rates of tubes S1→S4 and P4.
  - Disintegration rates are expected to fall approximately 10→100-fold from tubes S1→S3. This indicates that excess ${}^{32}$PO${}_{4}^{3-}$ is being washed out from the media.
  - Disintegration rates are expected to remain within the same order of magnitude between tubes S3 and S4. This indicates that all the excess ${}^{32}$PO${}_{4}^{3-}$ has been washed out.
  - Finally, the ratio of disintegration rates for P4:S4 is expected to be approximately 100:1. This ratio indicates the proportion of ${}^{32}$PO${}_{4}^{3-}$ firmly retained within the cytoplasm vs. the proportion of ${}^{32}$PO${}_{4}^{3-}$ released from the cytoplasm upon resuspension and centrifugation.

**Figure 2:**
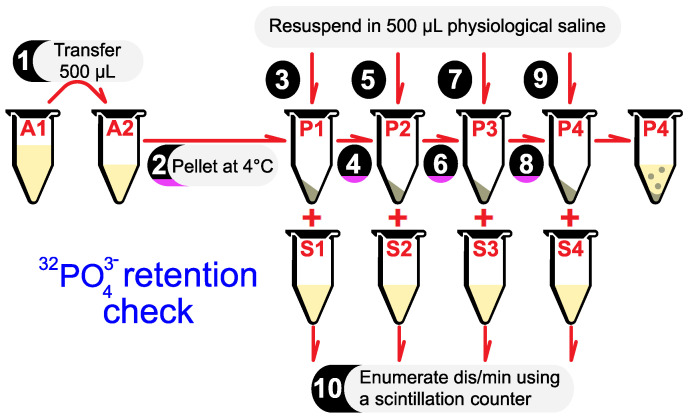
All the steps required to verify that ${}^{32}$PO${}_{4}^{3-}$ entered, and remains within, the bacterial cytoplasm.

All the steps described above are illustrated in Figure 2. Table 1 contains experimental values for all the variables discussed in this section.

### 3.3. Radiolabeled Phosphate Release

1. Transfer 333 μL of the suspension from tube P4 to a 50 mL centrifuge tube containing 9.667 mL saline, bringing the total volume to 10 mL.
2. Draw the entire contents (10 mL) into a 20 mL syringe.
3. Release 250 μL of the contents in the syringe into an empty microcentrifuge tube (Tube C). This tube serves as the pre-reaction total radiation check. The disintegration rate of this tube represents the total disintegration rate from ${}^{32}$PO${}_{4}^{3-}$ in both the cells and the saline medium.
4. Carefully remove and discard the needle. Attach a 0.2 μm syringe filter to the syringe. Attach a new needle to the syringe filter. The filter will separate the saline filtrate from cells, allowing for the measurement of ${}^{32}$PO${}_{4}^{3-}$ released from the cells while ignoring ${}^{32}$PO${}_{4}^{3-}$ still present within the cells.
5. Release 250 μL of the contents in the syringe into an empty microcentrifuge tube (tube T0). This tube’s baseline disintegration rate indicates the amount of ${}^{32}$PO${}_{4}^{3-}$ present in the saline medium (the filtrate) before the addition of your membrane disrupting agent (at time = 0).
6. Draw 250 μL of a pre-made stock solution of your membrane disrupting agent into the syringe. Note your stock solution will be diluted 40-fold within the syringe. Prepare your stock concentration accordingly. Replace your stock solution with saline for your negative control condition. Start timing your experiment from this point onwards.
7. At predetermined timepoints, release 250 μL of the contents in the syringe into microcentrifuge tubes (tubes T1→Tn).
8. Use a scintillation counter to enumerate the disintegration rates of tubes C, T0, T1→Tn. The percentage of ${}^{32}$PO${}_{4}^{3-}$ released at any timepoint (tube Tx) can be calculated using Equation (1).
  (1) $$\begin{array}{c} \begin{array}{c} Tx\left({\%}^{32}{\mathrm{PO}}_{4}^{3-}\mathrm{release}\right)=\frac{Tx(\mathrm{dis}/\min)-T0(\mathrm{dis}/\min)}{C(\mathrm{dis}/\min)-T0(\mathrm{dis}/\min)}\times 100 \end{array} \end{array}$$

**Figure 3:**
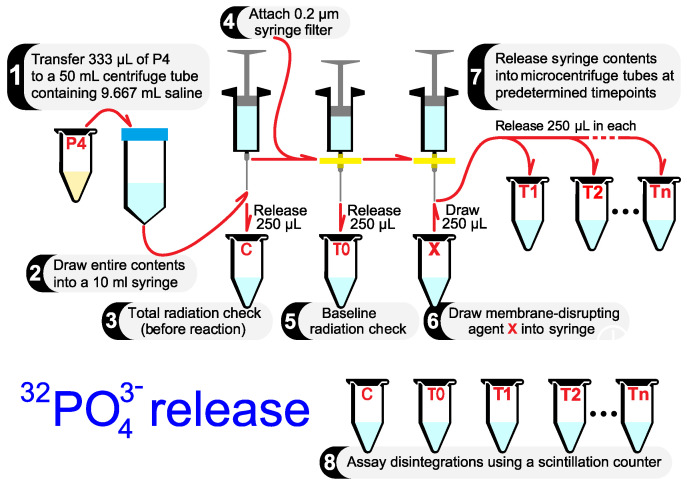
The ${}^{32}$PO${}_{4}^{3-}$ release assay. All the steps required to determine whether the chosen membrane-disrupting agent (agent X) disrupts membranes leading to the release of cytoplasmic small molecules, tracked using ${}^{32}$PO${}_{4}^{3-}$.

All the steps described above are illustrated in Figure 3. Table 1 contains experimental values for all the variables discussed in this section.

## 4. Expected Results

### 4.1. Rationale for the Development of the ${}^{32}$PO${}_{4}^{3-}$ Release Radioassay

We had previously developed the ${}^{32}$PO${}_{4}^{3-}$ release radioassay to understand the nature and kinetics of membrane disruption caused by Ω76, an antimicrobial peptide, against the cell membranes of *E. coli* (K-12 MG1655) and *A. baumannii* (P1270) [27]. The motivation for developing this radioassay arose from MBC assays, time-kill curves, mouse models, scanning electron microscopic experiments, and fluorescent confocal microscopic experiments performed on these organisms.

**Figure 4:**
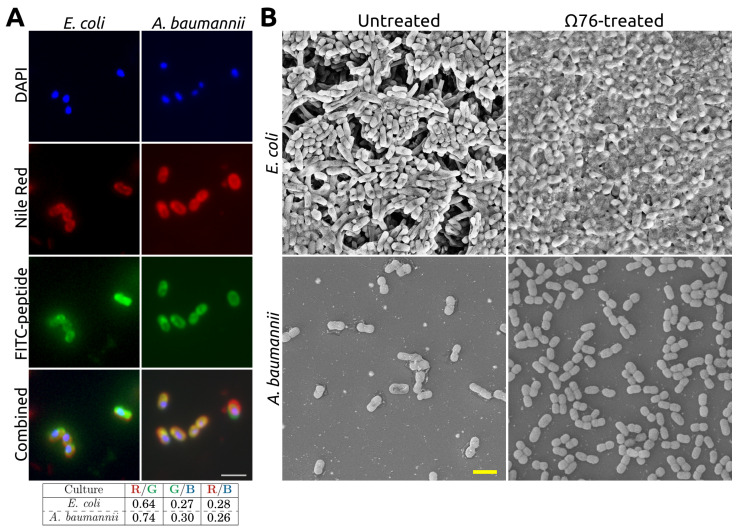
(**A**) Fluorescent confocal microscopy experiments for FITC-labeled Ω76 (8 μg/mL) against *E. coli* (K-12 MG1655) and *A. baumannii* (P1270). FITC-labeled Ω76 (green channel) was observed colocalizing with Nile red (red channel), which stains the cell membrane for both *E. coli* and *A. baumannii*. This was confirmed by Jaccard similarity coefficients of 0.64 and 0.74, respectively. FITC-labeled Ω76 (green channel) did not colocalize with DAPI (blue channel) that stains the bacterial chromosome for both *E. coli* and *A. baumannii*. This was confirmed by Jaccard similarity coefficients of 0.27 and 0.30, respectively. Therefore, Ω76 localizes within the cell membrane. Scale bar: 2 μm. The full method has previously been described in [27]. Note that the all images have been digitally magnified 3× after acquisition for clarity. (**B**) Scanning electron microscopy experiments for Ω76 (128 μg/mL) against *E. coli* (K-12 MG1655) and *A. baumannii* (P1270); Ω76 causes large-scale membrane disruptions and the release of cytoplasmic contents in *E. coli*. However, Ω76 causes no visible membrane disruptions on *A. baumannii*. Scale bar: 2 μm. The full method has previously been described in [27].

We noted that Ω76 possessed an MBC${}_{50}$ of 4 μg/mL against both *E. coli* and *A. baumannii* [27]; Ω76 is rapidly bactericidal, causing a ≥10${}^{5}$-fold reduction in *A. baumannii* c.f.u. counts over the course of 10 min [27]. Moreover, Ω76 displayed efficacy against *A. baumannii* in a mouse peritoneal model of infection, improving survival outcomes compared to controls [27]. Fluorescent, FITC-labeled Ω76 is incorporated into the cell membranes of both *E. coli* and *A. baumannii* (Figure 4A). However, upon treating *E. coli* and *A. baumannii* with Ω76, only *E. coli* displayed large-scale membrane disruption and the release of cytoplasmic contents (Figure 4B), while the cell membrane of *A. baumannii* appeared intact.

**Figure 5:**
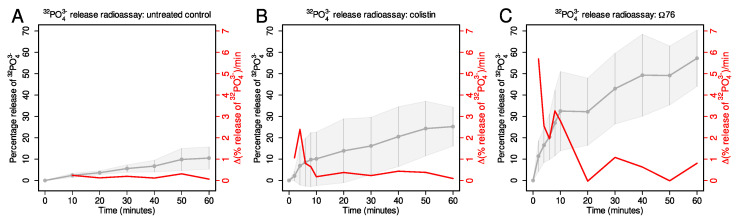
Results of the ${}^{32}$PO${}_{4}^{3-}$ release radioassay. Gray horizontal lines indicate mean ${}^{32}$PO${}_{4}^{3-}$ release at a given time (3 replicates). Gray vertical lines and the grey shaded area indicate standard deviation. Red lines (and red Y-axis) indicate the mean rate of ${}^{32}$PO${}_{4}^{3-}$ release per unit time (minute). Rates are calculated using a simple discrete first order differential of mean ${}^{32}$PO${}_{4}^{3-}$ release. *A. baumannii* (P1270) was used for all assays. (**A**) ${}^{32}$PO${}_{4}^{3-}$ release in the untreated condition; (**B**) ${}^{32}$PO${}_{4}^{3-}$ release from the colistin-treated condition (5 μg/mL); (**C**) ${}^{32}$PO${}_{4}^{3-}$ release from the Ω76-treated condition (32 μg/mL). Concentrations mimic those used in therapeutic doses. We reported the ${}^{32}$PO${}_{4}^{3-}$ release data in a previous study [27]. They have been reproduced here to aid in the description of our protocol.

Since Ω76 possesses in vitro and in vivo efficacy against *A. baumannii*, and since Ω76 is incorporated into the bacterial cell membrane, we hypothesized that Ω76 may cause nanoscale membrane disruptions (possibly with toroidal pore or barrel-stave architectures [29]) that are too small to be visualized using scanning electron microscopy. The results of the ${}^{32}$PO${}_{4}^{3-}$ release radioassay described below validated this hypothesis (Figure 5).

### 4.2. Expected Results for the ${}^{32}$PO${}_{4}^{3-}$ Release Radioassay

Nanoscale membrane disruptions are expected to cause the release of cytoplasmic small molecules into solution. The larger or more numerous the disruptions, the greater will be the release rate of these molecules. We had previously used ${}^{32}$PO${}_{4}^{3-}$ as a small molecule tracer to assay membrane disruption in *A. baumannii* under three conditions: untreated (negative control), colistin-treated (positive control), and Ω76 treated [19].

- The untreated condition displayed the least phosphate release. Only 10% of ${}^{32}$PO${}_{4}^{3-}$ was released after 60 min (Figure 5A). The rate of phosphate release remained fairly constant throughout this period, ranging from 0.06–0.3%/min.
- Colistin interacts with lipopolysaccharides (LPS) on both the outer and inner membranes, leading to membrane disruptions and the release of cytoplasmic contents [30]. A total of 25% of ${}^{32}$PO${}_{4}^{3-}$ was released after 60 min (Figure 5B). The rate of phosphate release peaked at 2.4%/min at t = 4 min.
- The Ω76 displayed the greatest phosphate release; 57% of ${}^{32}$PO${}_{4}^{3-}$ was released after 60 min. The rate of phosphate release peaked at 5.7%/min at t = 2 min. Therefore, Ω76 causes the release of a greater percentage of cytoplasmic ${}^{32}$PO${}_{4}^{3-}$, and at a higher rate, compared to both the untreated and colistin-treated conditions.

All the raw data for the experiments described above are provided in Table 1. It should be noted that a large variation in the initial disintegration rates for supernatant S1 is observed, with values ranging from 4.20 × 10${}^{6}$ to 1.34 × 10${}^{7}$. This occurs due to the short half-life of ${}^{32}$P (343 h). The amount of ${}^{32}$PO${}_{4}^{3-}$ present in the stock solution (and consequently supernatant S1) will rapidly decrease over time, which is especially noticeable for experiments performed over multiple days. This variation should not impact the experiment as the data is normalized later (Equation (1)).

These results, combined with our fluorescent microscopic and scanning electron microscopic observations, indicate that Ω76 causes nanoscale membrane disruptions that lead to the rapid loss of cytoplasmic contents and rapid bacterial death.

## 5. Discussion

The ${}^{32}$PO${}_{4}^{3-}$ release radioassay described here can provide information on both the ability of an agent to disrupt the bacterial cell membrane, as well as its membrane disruption kinetics. The motivation for developing this radioassay came from experiments performed on Ω76, an antimicrobial peptide. In vitro and in vivo experiments confirmed the peptide’s efficacy against *A. baumannii* (P1270) [27]. Fluorescent confocal microscopy experiments performed with FITC-labeled Ω76 confirmed that the peptide binds to the cell membrane of *A. baumannii* (Figure 4A). However, no membrane disruption was observed under scanning electron microscopy (Figure 4B). This leads us to conclude that Ω76 may act via the formation of nanometer-scale pores that cause the rapid exudation of small molecules from the cytoplasm, leading to bacterial death. This hypothesis was validated by the ${}^{32}$PO${}_{4}^{3-}$ release radioassay developed specifically for the task.

We used our radioassay to test small molecule leakage through the cell membrane of *A. baumannii* under three conditions: untreated, colistin-treated, and Ω76-treated. Untreated *A. baumannii* displayed minimal ${}^{32}$PO${}_{4}^{3-}$ leakage: 10% after 60 min with a peak ${}^{32}$PO${}_{4}^{3-}$ release rate of 0.3%/min (Figure 5A). Colistin-treated *A. baumannii* displayed moderate ${}^{32}$PO${}_{4}^{3-}$ leakage: 25% after 60 min with a peak ${}^{32}$PO${}_{4}^{3-}$ release rate of 2.4%/min (Figure 5B). Finally, Ω76-treated *A. baumannii* displayed extensive ${}^{32}$PO${}_{4}^{3-}$ leakage: 57% after 60 min with a peak ${}^{32}$PO${}_{4}^{3-}$ release rate of 5.7%/min (Figure 5C).

To the best of our knowledge, our protocol is the first to demonstrate the utility of ${}^{32}$PO${}_{4}^{3-}$ as a small molecule tracer to study membrane disruption. Conventionally, ${}^{51}$Cr is the radioisotope of choice for performing such assays [3]. However, working with ${}^{51}$Cr is less advantageous for several reasons: Firstly, ${}^{51}$Cr it is a γ-emitter [7]; γ-emitters present a greater hazard than β emitters, such as ${}^{32}$P. Working with ${}^{51}$Cr requires lead shielding and dose monitoring [8]. Secondly, ${}^{51}$Cr radioassays cannot measure kinetics, as they are limited to a single end-of-assay timepoint [2,3]. Thirdly, ${}^{51}$CrO${}_{4}^{2-}$ binds to bacterial lipopolysaccharides on the outer membrane, making it unsuitable for tracking the release of cytoplasmic contents through disruptions in the cell membrane [10]. The ${}^{51}$Cr assays are limited to measuring membrane disruption for eukaryotic cells, typically T-cells and natural killer (NK) cells [3].

Another novel aspect of the ${}^{32}$PO${}_{4}^{3-}$ release radioassay is that it can measure small molecule release kinetics in the order of minutes, and potentially seconds as well. In contrast, studies tracking membrane disruption kinetics using fluorescent dyes such as propidium iodide report time-intervals between successive readings in the order of hours [12,31,32]. This is due to a fundamental limitation of fluorescence assays: dyes such as propidium iodide and trypan blue cannot be used to track dying cells [33]. They interact with dead cells at a point in time too late to capture real-time membrane disruptions.

Although only demonstrated on the cell membrane of bacteria, our radioassay in principle can be adapted for use on any other membrane system. Leakage of small molecules from large unilamellar lipid vesicles can be assayed simply and directly using the ${}^{32}$PO${}_{4}^{3-}$ release radioassay, in contrast to indirect and complicated methods such as fluorescent correlation spectroscopy [34]. Our radioassay can be used to quantify changes in the membrane permeability of animal cells that can occur during a viral infection [35,36], interaction with pore-forming toxins [37,38], or during apoptosis [39,40]. Our radioassay can also be used to easily study mitochondrial permeability transitions in isolated mitochondria [41,42], which involve the sudden and rapid efflux of low molecular weight solutes.

It should be noted that radioassays have inherent drawbacks compared to fluorescent assays. Working with radioactive material requires special handling facilities that may not be available to all researchers. Handling radioisotopes requires appropriate PPE [28] and safety precautions not associated with fluorescent assays. Although${}^{32}$PO${}_{4}^{3-}$ is affordable, due to its low half-life (343 h), all experiments must be performed within a few weeks of acquiring the material. Alternatively, ${}^{32}$PO${}_{4}^{3-}$ must be continuously ordered.

Despite these drawbacks, we expect the protocol described here to be of use to bacteriologists as well as researchers in any other field who study the mechanisms of membrane disruption.

## Figures and Tables

**Table 1:** Raw data for the results of the ${}^{32}$PO${}_{4}^{3-}$ release radioassay illustrated in Figure 5. Three replicates per condition were performed, and the data for each replicate is provided in the columns rep-1→rep-3. All values are in disintegrations/min.

	Tube	Untreated Control			Colistin			Ω76		
		**Rep-1**	**Rep-2**	**Rep-3**	**Rep-1**	**Rep-2**	**Rep-3**	**Rep-1**	**Rep-2**	**Rep-3**
	S1	1.25 $\times \;10^{7}$	1.18 $\times \;10^{7}$	8.56 $\times \;10^{6}$	1.30 $\times \;10^{7}$	5.49 $\times \;10^{6}$	4.20 $\times \;10^{6}$	1.34 $\times \;10^{7}$	2.29 $\times \;10^{7}$	7.00 $\times \;10^{6}$
	S2	4.88 $\times \;10^{6}$	3.17 $\times \;10^{5}$	3.44 $\times \;10^{5}$	4.30 $\times \;10^{6}$	6.80 $\times \;10^{5}$	9.51 $\times \;10^{4}$	3.27 $\times \;10^{5}$	5.32 $\times \;10^{5}$	4.57 $\times \;10^{5}$
	S3	1.92 $\times \;10^{4}$	6.73 $\times \;10^{3}$	1.05 $\times \;10^{4}$	1.90 $\times \;10^{4}$	6.55 $\times \;10^{4}$	1.15 $\times \;10^{4}$	4.74 $\times \;10^{4}$	2.12 $\times \;10^{4}$	2.53 $\times \;10^{4}$
	S4	1.05 $\times \;10^{4}$	4.38 $\times \;10^{3}$	4.05 $\times \;10^{3}$	1.03 $\times \;10^{4}$	9.79 $\times \;10^{3}$	4.08 $\times \;10^{3}$	1.51 $\times \;10^{4}$	1.10 $\times \;10^{4}$	1.15 $\times \;10^{4}$
	P4	1.78 $\times \;10^{6}$	5.32 $\times \;10^{5}$	6.67 $\times \;10^{5}$	1.55 $\times \;10^{6}$	2.00 $\times \;10^{6}$	3.46 $\times \;10^{5}$	1.77 $\times \;10^{6}$	5.96 $\times \;10^{5}$	1.87 $\times \;10^{6}$
	C	3.23 $\times \;10^{4}$	1.05 $\times \;10^{4}$	1.21 $\times \;10^{4}$	3.35 $\times \;10^{4}$	5.85 $\times \;10^{4}$	1.70 $\times \;10^{4}$	3.33 $\times \;10^{4}$	1.16 $\times \;10^{4}$	5.80 $\times \;10^{4}$
0 m	T0	4.65 $\times \;10^{2}$	3.26 $\times \;10^{2}$	2.71 $\times \;10^{2}$	6.39 $\times \;10^{2}$	1.05 $\times \;10^{3}$	1.78 $\times \;10^{2}$	1.30 $\times \;10^{3}$	4.96 $\times \;10^{2}$	1.58 $\times \;10^{3}$
2 m	T1				1.07 $\times \;10^{3}$	1.27 $\times \;10^{3}$	9.63 $\times \;10^{2}$	7.31 $\times \;10^{3}$	1.72 $\times \;10^{3}$	4.09 $\times \;10^{3}$
4 m	T2				1.67 $\times \;10^{3}$	1.29 $\times \;10^{3}$	3.05 $\times \;10^{3}$	8.91 $\times \;10^{3}$	2.49 $\times \;10^{3}$	6.00 $\times \;10^{3}$
6 m	T3				1.86 $\times \;10^{3}$	1.36 $\times \;10^{3}$	3.75 $\times \;10^{3}$	1.15 $\times \;10^{4}$	2.54 $\times \;10^{3}$	7.73 $\times \;10^{3}$
8 m	T4				2.16 $\times \;10^{3}$	1.37 $\times \;10^{3}$	4.23 $\times \;10^{3}$	1.51 $\times \;10^{4}$	3.27 $\times \;10^{3}$	8.72 $\times \;10^{3}$
10 m	T5	1.48 $\times \;10^{3}$	4.58 $\times \;10^{2}$	5.94 $\times \;10^{2}$	2.31 $\times \;10^{3}$	1.59 $\times \;10^{3}$	4.27 $\times \;10^{3}$	1.82 $\times \;10^{4}$	3.60 $\times \;10^{3}$	1.10 $\times \;10^{4}$
20 m	T6	1.93 $\times \;10^{3}$	6.18 $\times \;10^{2}$	6.76 $\times \;10^{2}$	3.69 $\times \;10^{3}$	2.07 $\times \;10^{3}$	5.31 $\times \;10^{3}$	1.69 $\times \;10^{4}$	3.90 $\times \;10^{3}$	1.13 $\times \;10^{4}$
30 m	T7	2.78 $\times \;10^{3}$	7.17 $\times \;10^{2}$	9.38 $\times \;10^{2}$	5.55 $\times \;10^{3}$	3.04 $\times \;10^{3}$	5.23 $\times \;10^{3}$	2.08 $\times \;10^{4}$	4.95 $\times \;10^{3}$	1.74 $\times \;10^{4}$
40 m	T8	3.60 $\times \;10^{3}$	8.37 $\times \;10^{2}$	8.96 $\times \;10^{2}$	7.32 $\times \;10^{3}$	4.85 $\times \;10^{3}$	5.98 $\times \;10^{3}$	2.38 $\times \;10^{4}$	5.45 $\times \;10^{3}$	2.02 $\times \;10^{4}$
50 m	T9	5.48 $\times \;10^{3}$	9.51 $\times \;10^{2}$	1.17 $\times \;10^{3}$	8.81 $\times \;10^{3}$	7.51 $\times \;10^{3}$	6.35 $\times \;10^{3}$	2.12 $\times \;10^{4}$	6.17 $\times \;10^{3}$	2.11 $\times \;10^{4}$
60 m	T10	5.71 $\times \;10^{3}$	1.09 $\times \;10^{3}$	1.16 $\times \;10^{3}$	8.83 $\times \;10^{3}$	1.04 $\times \;10^{4}$	5.96 $\times \;10^{3}$	2.42 $\times \;10^{4}$	6.54 $\times \;10^{3}$	2.74 $\times \;10^{4}$

## Data Availability

All data has been made available within this paper.

## Sample Availability

Samples of Ω76 are available from the authors.
